# How do digital inclusive programs promote healthy aging: The case of Hong Kong

**DOI:** 10.1093/geroni/igaf122.1745

**Published:** 2025-12-31

**Authors:** Bobo Hi Po Lau, Alex Kin-shing Chan, Gigi Lam, Alex Chi-keung Chan, Rick Yiu-cho Kwan, Daniel Dick-man Leung, Alex Pak-ki Kwok, Fan Lui

**Affiliations:** Hong Kong Shue Yan University, Hong Kong, China (People’s Republic); Hong Kong Shue Yan University, Hong Kong, China (People’s Republic); Hong Kong Shue Yan University, Hong Kong, China (People’s Republic); Tung Wah College, Tsim Sha Tsui, Hong Kong, China (People’s Republic); Tung Wah College, Tsim Sha Tsui, Hong Kong, China (People’s Republic); Tung Wah College, Tsim Sha Tsui, Hong Kong, China (People’s Republic); The Chinese University of Hong Kong, Hong Kong, China (People’s Republic); Hong Kong Shue Yan University, Hong Kong, China (People’s Republic)

## Abstract

Metropolises worldwide have adopted community-based support for older adults to foster digital inclusiveness, thereby facilitating their well-being and societal engagement. Hong Kong ranks seventh on the IMD Digital Competitiveness Index in 2024. The government has been funding community-based digital support targeting older adults since early 2010. This study content-analysed 39 digital inclusiveness programs for older adults between 2013 and 2025 under five key government schemes. We coded their content by the digital divide levels, social and intermediary determinants of health, and aspects of healthy aging they affected. Our findings show that 20.5% of the programs enhanced material access through renting or lending smart devices to older adults; 59.0% facilitated device operation skills, 84.6% taught strategic skills (e.g., telehealth, cognitive training), and 35.9% exposed older adults to devices beyond smartphones including wearable health devices and aerial cameras. These programs fostered health management (20.5%) and social service utilization (15.4%), thereby enhanced healthy aging domains including health and cognition (33.3%), psychological and spiritual well-being (25.6%), and community engagement (17.9%). Another survey of our team with 491 participants aged 60 or older found older adults use smart technologies for only 3.1 life domains (‘communication’ (82.7%), ‘economics’ (13.2%), and ‘daily practical’ (12.2%)); usage variety is constrained by their age and education. As device ownership among older adults had peaked in most metropolises post-pandemic, the digital divide shifted to be one related to the extensiveness and flexibility of usage. Our findings will illuminate areas of technology usage that require additional input for enhancing digital inclusiveness.

